# Moving Online

**DOI:** 10.1093/tas/txaa035

**Published:** 2020-04-06

**Authors:** J Scott Radcliffe

**Affiliations:** Department of Animal Sciences, Purdue University, West Lafayette, IN

## INTRODUCTION

In the midst of the COVID-19 global pandemic, classes worldwide are instantly being converted to online classes. If people are unfamiliar with online teaching technologies, this can be a difficult transition. This review focuses on 1) making the best of a teaching “triage” situation, how can we leverage our strengths and still quickly provide quality to our students, 2) a review of synchronous and asynchronous online teaching options, 3) a review of interactive teaching options, and 4) a review of testing changes and options. Please note, this video review is not designed to be an in-depth scientific review of teaching methods, but a review of immediate options to help move animal science classes online.

